# P2X7 receptor knockout does not alter renal function or prevent angiotensin II-induced kidney injury in F344 rats

**DOI:** 10.1038/s41598-024-59635-x

**Published:** 2024-04-26

**Authors:** Josselin Nespoux, Marie-Louise T. Monaghan, Natalie K. Jones, Kevin Stewart, Laura Denby, Alicja Czopek, John J. Mullins, Robert I. Menzies, Andrew H. Baker, Matthew A. Bailey

**Affiliations:** Edinburgh Kidney, British Heart Foundation Centre for Cardiovascular Science, The University of Edinburgh, Edinburgh, UK

**Keywords:** Cardiovascular diseases, Kidney

## Abstract

P2X7 receptors mediate immune and endothelial cell responses to extracellular ATP. Acute pharmacological blockade increases renal blood flow and filtration rate, suggesting that receptor activation promotes tonic vasoconstriction. P2X7 expression is increased in kidney disease and blockade/knockout is renoprotective. We generated a P2X7 knockout rat on F344 background, hypothesising enhanced renal blood flow and protection from angiotensin-II-induced renal injury. CRISPR/Cas9 introduced an early stop codon into exon 2 of *P2rx7*, abolishing P2X7 protein in kidney and reducing *P2rx7* mRNA abundance by ~ 60% in bone-marrow derived macrophages. The M1 polarisation response to lipopolysaccharide was unaffected but P2X7 receptor knockout suppressed ATP-induced IL-1β release. In male knockout rats, acetylcholine-induced dilation of the renal artery ex vivo was diminished but not the response to nitroprusside. Renal function in male and female knockout rats was not different from wild-type. Finally, in male rats infused with angiotensin-II for 6 weeks, P2X7 knockout did not reduce albuminuria, tubular injury, renal macrophage accrual, and renal perivascular fibrosis. Contrary to our hypothesis, global P2X7 knockout had no impact on in vivo renal hemodynamics. Our study does not indicate a major role for P2X7 receptor activation in renal vascular injury.

## Introduction

Adenine and uridine nucleotides can be released from the cells to exert potent paracrine effects on cell function through activation of cognate P2 (purinergic) receptors on the cell membrane^1,2^. These receptors are either ion channels gated by ATP (P2X) or G-protein coupled receptors (P2Y), activated by ATP, ADP, UTP or UDP^1,2^. The kidney expresses several P2X and P2Y subtypes and purinergic signalling regulates many aspects of kidney physiology, including renal hemodynamics^3^, epithelial cell metabolism and tubular transport^4^. Extracellular ATP is also recognized as a danger activated molecular pattern and in experimental animals, abnormal purinergic signalling contributes to kidney disease processes such as salt retention/hypertension^5^, sustained inflammation, fibrosis and tissue remodelling^6^.

**Figure 1:**
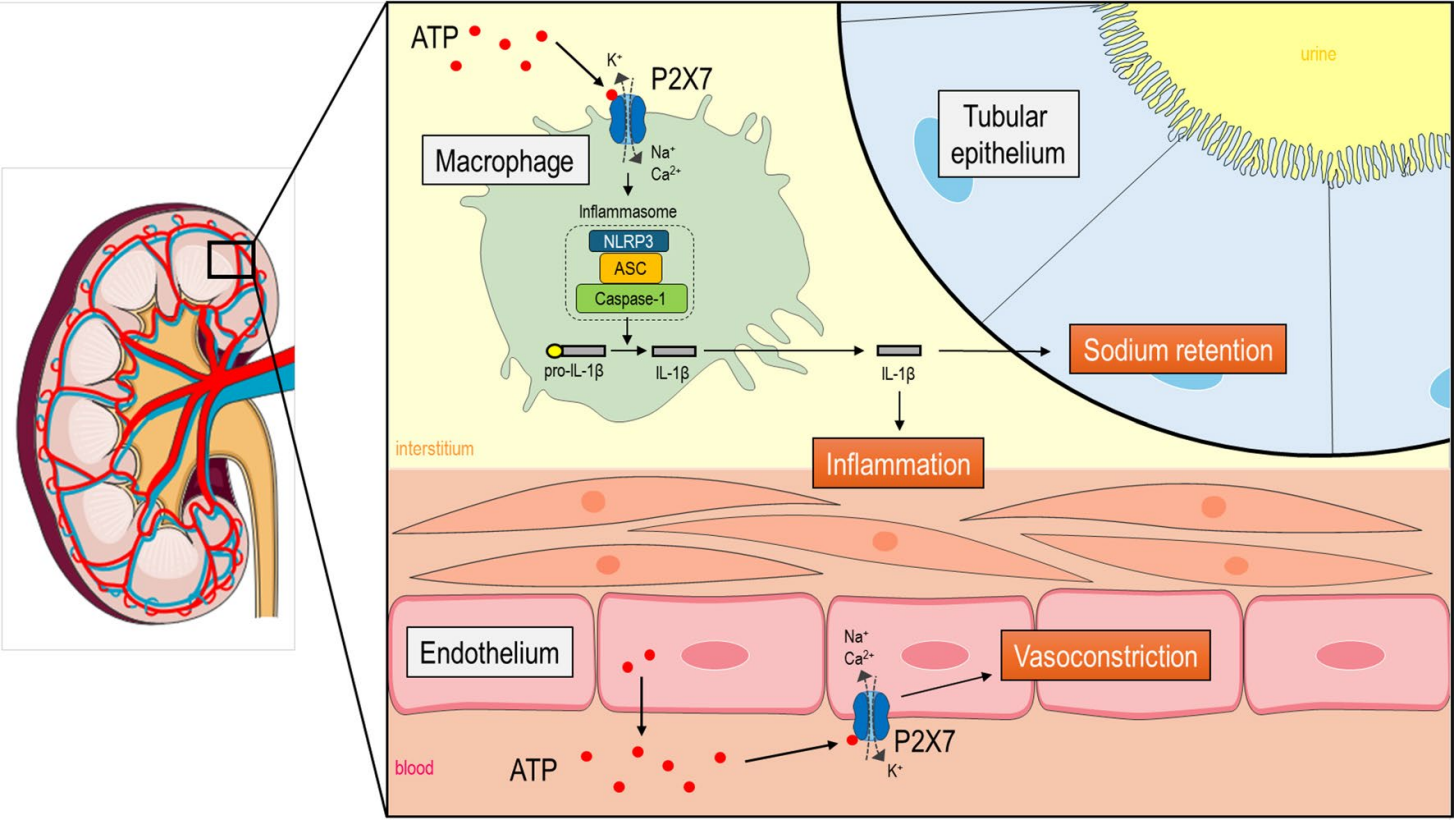
The role of P2X7 receptors in the hypertensive kidney. P2X7 receptors are non-selective cation channel receptors activated by extracellular adenosine triphosphate (ATP). P2X7 receptors are abundantly expressed by immune cells, such as macrophages. ATP released by damaged or activated cells activates P2X7 receptors on macrophages to mediate NOD−, LRR− and pyrin domain-containing protein 3 (NLRP3), Apoptosis-associated speck-like protein containing a caspase recruitment domain (CARD) (ASC) and caspase-1 assembly leading to inflammasome activation, and the subsequent maturation and release of pro-inflammatory cytokines such as interleukin (IL)-1β. These cytokines promote renal interstitial inflammation and promote sodium retention by tubular epithelial cells, thereby contributing to salt-sensitivity and hypertension. P2X7 receptors are also expressed in renal vascular endothelial cells, particularly in the pre-glomerular vasculature and *vasa recta*. ATP, released by endothelial cells, can activate endothelial P2X7 receptors in an autocrine/paracrine fashion to promote vasoconstriction, ultimately decreasing renal blood flow (RBF) and glomerular filtration rate (GFR). Figure contains a modified illustration from Servier Medical Art. Servier Medical Art by Servier is licensed under a Creative Commons Attribution 4.0 Unported License (https://creativecommons.org/licenses/by/4.0/).

This study focuses on the P2X7 receptor, which is expressed in myeloid cells and mediates the ATP-induced activation of the NLRP3 inflammasome and release of inflammatory cytokines IL-1β and IL-18^7,8^ (Fig. 1). P2X7 receptor mRNA abundance is higher in peripheral blood mononuclear cells of kidney disease patients than in healthy controls^9^. In the kidney itself, the normally low expression of P2X7 is substantially increased in biopsies from people with diabetic and non-diabetic kidney disease^10–12^. A similar induction of P2X7 receptor expression is observed in experimental kidney disease and hypertension^11,13–15^. In a rat model of nephrotoxic nephritis, in which high intrarenal levels of IL-1β drive glomerular injury, pharmacological P2X7 receptor blockade is beneficial; P2X7 knockout mice were similarly protected^16^. Renoprotection was associated with reduced accumulation of macrophages within the glomerulus and was attributed to a direct anti-inflammatory effect of P2XR7 antagonism.

However, *P2rx7* mRNA is also highly expressed by vascular endothelial cells under normal, noninflammatory conditions^17^ (Fig. 1). In kidney, P2X7 immunolocalizes to the endothelium of the preglomerular arteries, the afferent arteriole and to *vasa recta*^5^. Functional data are limited but in ex vivo kidney slices, the P2X7-selective agonist BzATP induces contraction of *vasa recta* pericytes^18^. In rats treated chronically with angiotensin II, P2X7 blockade increases excretory output^14^, improves medullary blood flow and oxygenation^13^ and increases glomerular filtration rate (GFR) by predominantly reducing afferent arteriole resistance^15^. Thus, P2X7 receptor activation influences immune cell function and migration in disease. Receptor activation also appears to exert a tonic vasoconstriction, particularly when arterial tone is high (Fig. 1). Here, we used CRISPR/Cas9 to delete P2X7 receptors in F344 rats to generate a tool for detailed physiological and disease-modelling studies. Also, we closely examined renal function in female rats as previous studies had focused only on male knockout animals. Under control conditions, P2X7 receptor knockout reduced nitric oxide-dependent vasodilation of the renal artery in males only, but did not alter renal hemodynamics or tubular sodium handling in vivo. Chronic angiotensin II infusion induced albuminuria, tubular injury, renal inflammation and perivascular fibrosis with no evidence of renal protection in P2X7 receptor knockouts.

## Methods

### Animals and ethical review

Methods are reported in accordance with ARRIVE guidelines (https://arriveguidelines.org) for the reporting of animal experiments. The animal experiments were conducted under a UK Home Office Project License P1D9E2AD5 (holder: M.A.B.) and in accordance with the UK Home Office Animals (Scientific Procedures) Act of 1986 after approval by the Animal Welfare and Ethical Review Board of The University of Edinburgh.

All rats used were housed in the same holding room under controlled temperature (21 ± 1 °C), humidity (55 ± 10%) and light/dark cycle (light 7:00 a.m.–7:00 p.m.). Rats had ad libitum access to water and a standard rodent chow (RM1, 0.25% Na, Cat. No: 801151, SDS Diets, UK) throughout.

### Generation of the P2X7 KO rat

A global P2X7 KO on a F344/IcoCrl background was created using CRISPR/Cas9 technology and denominated F344.*P2rx7*^*em1UoE*^. For simplification purposes, F344.*P2rx7*^*em1UoE*^ is referred to as *P2rx7*^*−/−*^ in this manuscript. The dual expression plasmid px330 (Addgene, Watertown, MA) containing the guide RNA (gRNA) sequence (GTGTGCACAGAGCTGATAAG) was made by the Gene Editing Rat Resource Center at the Medical College of Wisconsin and was given free of charge to RIM. In Edinburgh, the plasmid (5 ng/μL) was injected into pronuclei of single cell F344/IcoCrl rat embryos, which were transferred into the oviduct of pseudopregnant rats. The construct was a dual expression plasmid expressing the gRNA by a U6 promoter, and the Cas9 by a cytomegalovirus. The targeted 20 bp sequence localized within exon 2 of *P2rx7* gene was substituted with a 22 bp sequence (CTTAAATCAGCTCTGTGCACAC) corresponding to a two-adenine insertion predicted to introduce a premature termination codon. To screen for the presence of the mutation in the offspring, genomic DNA was extracted from ear clippings and polymerase chain reaction was carried out. The 2 bp insertion in exon 2 was confirmed by the DNA Sequencing Services at the University of Dundee, using Sanger analysis.

### Isolation and culture of bone marrow-derived macrophages (BMDM)

Adult male and female *P2rx7*^−/−^ and WT rats (4–6 months old) were culled by CO_2_ asphyxiation and femurs and tibias excised, all between 8:00 a.m. and 10:00 a.m. All soft tissue was removed, and bones were transferred to a primary tissue culture sterile hood and cleaned in 70% ethanol. Both ends of the cleaned bones were cut using sterile tools and bone marrow cells were flushed into a 50 mL Falcon tube using a 20-gauge needle attached to a syringe containing 10 mL warm Dulbecco’s phosphate buffer saline (DPBS; Life Technologies). Tubes were centrifuged at 1000G for 5 min at RT.

Cells were re-suspended in 10 mL warm (37 °C) culture media consisting of Dulbecco's modified eagle medium/nutrient mixture F-12 (DMEM/F-12, GlutaMAX™ supplement; Gibco, Cat. No: 10565018) supplemented with 10% heat-inactivated foetal bovine serum (FBS) (Gibco, Cat. No: 10082147), 1% penicillin and streptomycin, and containing 10 ng/mL recombinant rat macrophage colony-stimulating factor (rM-CSF, Peprotech, Cat. No: 400-28). Cell suspensions from all bones of a given rat were pooled (total of 40 mL) and transferred to a 60 mL standard perfluoroalkoxy alkanes jar (Qmx Laboratories Ltd, Essex, UK). Cells were cultured in suspension for 7 days at 37 °C, 5% CO_2_. On day 2 and 4, 20 mL of media was carefully pipetted off and replaced with fresh culture media. On day 7, ~ 30 mL of media were discarded and the remaining 10 mL were used to gently resuspend the cells using up-and-down pipetting with a 10 mL stripette. Cell suspensions were centrifuged at 1000G for 5 min at RT and cell pellets were resuspended in fresh culture media. Cells were counted using a BioRad TC10 automated cell counter in order to seed 2 million cells per well, in a total of 2 mL culture media, in standard 6-well plates (Corning, USA). Cells were incubated for 24 h at 37 °C, 5% CO_2_ which allowed finalizing macrophage differentiation and attaching to the bottom of the wells.

### Stimulation of BMDM

BMDM were stimulated with 1 µg/mL lipopolysaccharide (LPS, from *E.Coli*, Sigma, Cat. No: LPS25) for 4 h, followed by 3 mM ATP (Sigma, Cat. No: A6419) for 1 h. Cell supernatants were collected and stored at -80 °C. The stable NO metabolite nitrite (NO_2_^-^) concentrations were determined in freshly collected BMDM supernatants using Griess reagent system according to the manufacturer's instructions (G2930, Promega, Fitchburg, WI, USA). Secreted IL‐1β in supernatant was measured using a sandwich ELISA kit (R&D, Cat. No: RLB00) according to the manufacturer's instructions.

### Reverse transcription and real-time PCR

Total RNA from either ~ 30 mg piece of frozen kidney cortex or BMDM was isolated using the RNeasy Plus Mini Kit (Qiagen, Germantown, MD, USA) and cDNA was prepared using the High-Capacity RNA-to-cDNA Reverse Transcription kit (Applied Biosystems, CA, USA). Quantitative polymerase chain reaction (qPCR) was performed in triplicate with the Roche Universal Probe Library (UPL) system using a Lightcycler 480 (Roche, West Sussex, UK). For each gene, a 7-point standard curve (1:10–1:640) was generated using a mix of all cDNA samples to be analyzed to which each sample was normalized. Kidney and BMDM cDNA samples were diluted 1:40 in nuclease free water prior to qPCR. Primers for rat *Actb*, *Il1b*, *Il6*, *P2rx4*, *P2rx7*, *Tbp* and *Tnf* (Supplementary Table 1) were designed with the UPL Assay Design Centre and used at a final concentration of 250 nM with UPL probes (Supplementary Table 1) and PerfeCTa FastMix II (Quanta bio, Beverly, MA, USA). Four different primer sets were used to detect *P2rx7* mRNA abundance. Absolute quantification was used to determine each gene expression level and results were normalized to that of the housekeeping gene (*Actb* or *Tbp*).

### Kidney protein extraction and Western blotting

Snap frozen kidney cortex tissue was homogenized in ice-cold RIPA buffer (Cat. No: 39244, SERVA Electrophoresis GmbH, Germany) with Complete protease inhibitor cocktail tablet (Roche, UK) and phosphatase inhibitors cocktail 3 (P0044-1ML, Sigma-Aldrich, UK) using a Tissue Lyser II (Qiagen, UK). Protein concentration was quantified using the Pierce BCA Assay kit according to manufacturer’s instructions (Cat. No: 23225, Thermo Fisher Scientific, UK). Lysates at 20 μg/lane and protein ladder (Amersham rainbow ladder, Thermo Fisher Scientific, UK) were resolved on a Bolt 4–12% Bis–Tris Plus pre-cast gel (Invitrogen, Carlsbad, CA, USA). Gel proteins were transferred to nitrocellulose membranes (Nitrocellulose/Filter Paper Sandwich, 0.2 μm, Invitrogen) and immunoblotted with the rabbit-anti-rat P2X7 primary antibody (APR-004, 1:500, Alomone Labs, Jerusalem, Israel) and mouse-anti-rat GAPDH (Ab8245, 1:1000, Abcam, Cambridge, UK) overnight. Then, membrane was incubated with secondary antibodies IRdye 800CW goat-anti-rabbit (Cat. No: 926-32211, 1:10,000, LI-COR, Lincoln, NE, USA) and IRdye 700CW donkey-anti-mouse (Cat. No: 926-68072, 1:10,000, LI-COR, Lincoln, NE, USA). Membrane was visualized using the LI-COR Odyssey clx.

### Ex vivo wire myography

Animals were culled between 8:00 a.m. and 10:00 a.m. for artery collection. ~ 2 mm segments of a main renal artery were mounted in a multi-myography system (Cat. No: 610 M, Danish Myo Technology) containing physiological salt solution (119.0 mM NaCl, 4.7 mM KCl, 2.5 mM CaCl_2_, 1.2 mM MgSO_4_, 25.0 mM NaHCO_3_, 1.2 mM KH_2_PO_4_, 27.0 μM EDTA, 5.5 mM d-glucose) aerated at 37 °C with 95% O_2_/5% CO_2_. In all studies, the viability of each artery was first confirmed by a contractile response on addition of a high-potassium physiological salt solution (KPSS; 4.7 mM NaCl, 119.0 mM KCl, 2.5 mM CaCl_2_, 1.2 mM MgSO_4_, 25.0 mM NaHCO_3_, 1.2 mM KH_2_PO_4_, 27.0 μM EDTA, 5.5 mM d-glucose), repeated three times. Alpha-adrenergic-mediated vasoconstriction was assessed by exposing vessels to cumulative concentrations (1 nM–3 μM) of phenylephrine (PE). To assess vasodilator capacity, vessels were preconstricted with half-maximal effective concentration (EC_50_) of PE, and cumulative concentration curves (1 nM–3 μM) were obtained to acetylcholine (ACh) and sodium nitroprusside (SNP). A 30-min washout was allowed between drugs. For vasoconstriction, the maximum contraction values (in mN) induced by each PE concentrations were normalised to the maximum contraction induced by KPSS at the start (%KPSS). For vasodilation, the minimum tension values (in mN) measured for each ACh or SNP concentrations were normalised to the initial contraction force induced by PE-preconstriction before the start of vasodilation drugs application (% of PE preconstriction).

### In vivo kidney function and acute pressure natriuresis study

The effects of P2X7 KO on blood pressure (BP), renal hemodynamics and tubular handling of electrolytes were assessed in 3.5-to-4.5-month male and female rats under non-recovery anaesthesia (sodium thiopental; 50 mg/kg in 0.9% NaCl saline i.p.; MercuryPharma, London, UK). Two rats were used per day and genotypes mixed up. Surgeries and kidney function parameters measurements were performed by J.N. and K.S. between ~ 11:00 a.m. and ~ 2:30 p.m0 (rat 1), and between ~ 3:00 p.m. and ~ 6:30 p.m. (rat 2). The right jugular vein was cannulated to infuse a saline solution (pH = 7.4, 1 mL/h/100 g BW) supplemented with 2% bovine serum albumin (w/v) to limit extravasation, and 0.25% fluorescein isothiocyanate (FITC)-inulin (Cat. No: F3272-1G, Sigma) for GFR determination. A tracheotomy was performed, and the right carotid artery cannulated with p50 polyethylene tubing (Smiths Medical International Ltd, Hythe, UK) pre‐flushed with heparinised saline. The arterial line was connected to a calibrated BP transducer and multi‐channel data acquisition system (Powerlab; ADInstruments, Oxford, UK) for real‐time BP recording. Bladder was catheterized with p50 polyethylene tubing for urine collection. A laparotomy was performed, and infusion rate was increased to 2 mL/h/100 g BW to minimize surgical fluid losses. Loose ligatures were placed around the coeliac and superior mesenteric artery, as well as the aorta distally to renal arteries branching. A Doppler ultrasound probe (Cat. No: MA1PRB, Transonic, USA) was placed around the main renal artery and ultrasound gel was used for acoustic coupling for real-time renal blood flow (RBF) measurement. To estimate medullary blood perfusion, a needle laser Doppler flowmetry probe (Cat. No: MNP110XP; ADInstruments, UK) was inserted into the right kidney, 4–5 mm from dorsal surface, orientated toward the renal hilus. A second needle probe was placed at the surface of the kidney to estimate cortical blood perfusion.

After a post‐surgical equilibration period of ∼ 30 min, parameters were measured over a 30-min baseline period. Acute pressure natriuresis was then induced by sequential ligation of the coeliac and cranial mesenteric arteries, followed by the distal aorta, as described previously^14,19,20^. After each ligation, urine was collected, and parameters measured over a 30-min period. The increase in BP after the first ligation generally failed to maintain over the 30-min period, whereas BP reached a plateau after ligation 2. Thus, data from baseline and following the second ligation were used for analyses. Data are presented as the percentage change from baseline for each rat, unless stated otherwise.

### Blood pressure measurement in conscious rats

Radiotelemetry devices (Cat. No: HD-s10; Data Sciences International, Hertogenbosch, Netherlands) were implanted into male WT and *P2rx7*^*−/−*^ rats under anaesthesia (4% isoflurane for induction, 2–3% for maintenance) using aseptic techniques. The sensor tip was inserted and secured into the abdominal aorta, and, after surgery, rats were allowed to recover in a heated box. While under anaesthesia, the animals received analgesic (Buprenorphine, 0.05 mg/kg) via subcutaneous injection. The animals were also given 1 mL of 0.9% sterile saline (s.c.) to aid in recovery. For 48 h post-surgery, rats were given buprenorphine jelly (0.5 mg/kg). After 1 week of recovery, systolic BP (SBP) and diastolic BP (DBP) data were acquired at 1 kHz over a 1 min period in every hour for a period of five consecutive days. Zeitgeber time zero (ZT = 0) was defined as the start of the light period at local time 7:00 a.m. Data of each day were averaged to obtain a representative 24 h-period BP recording. Data from both light and dark 12-h periods were compared for assessment of diurnal rhythmicity.

### Chronic ANGII infusion study

3-to-5-month male WT and *P2rx7*^*−/−*^ rats were anesthetised with 3% isoflurane inhalation in 100% O_2_, and an osmotic minipump (Alzet 2006, Durect, Cupertino, CA) was implanted subcutaneously using aseptic techniques. ANGII was infused at a rate of 250 ng/kg/min for ~ 6 weeks. Naive age-matched rats were used as controls. Preliminary experiments revealed that ANGII infusion was poorly tolerated by F344 rats, as shown by signs of illness including body weight loss, loss of grooming, reluctance to move, and abnormal posture. Following advice of our veterinary team, blood pressure was not recorded to avoid excessive handling of the animals that may cause stress, and mashed food was provided throughout the entire experiment to facilitate feeding and avoid excessive body weight loss. At the end of ANGII infusion period, rats were culled by CO_2_ asphyxiation. Bladder urine was collected for urine analyses, and kidney and heart were weighed and processed for histological analyses. Tibia length was measured using a digital calliper.

### Plasma and urine analyses

Plasma sodium (Na^+^), potassium (K^+^) and chloride (Cl^−^) concentrations were measured simultaneously in undiluted samples by ion-selective electrode (9180 electrolyte analyser, Roche diagnostics Ltd). Urine electrolytes were measured in samples diluted 1:1 using a Spotchem EL SE-1520 electrolyte analyser with ion-selective electrodes (Arkray Inc., Kyoto, Japan). Urine nitrite/nitrate (NOx) were quantified in samples diluted 1:20 using a colorimetric assay based on nitrate reductase activity and Griess reaction (Cat. No: ADI-917-010, Enzo Life Science, Farmingdale, NY, USA). Urine creatinine measurements were determined using a creatininase/creatinase enzymatic method^21^ making use of a commercial kit (Sentinel Diagnostics via Alpha Laboratories Ltd., Eastleigh, UK) adapted for use on a Cobas Fara analyser (Roche Diagnostics Ltd, Welwyn Garden City, UK). Urine albumin measurements were determined using a commercial kit (DiaSys Diagnostic Systems, Germany) adapted for use on a Cobas Mira analyser (Roche Diagnostics Ltd, Welwyn Garden City, UK). This immunoturbidimetric assay was standardised against purified mouse albumin standards (Sigma Aldrich, Poole, UK) with samples diluted in deionised water as appropriate. Urine albumin-to-creatinine ratios (UACR) were calculated and presented in milligram of albumin per millimole of creatinine (mg/mmol). Urine KIM-1 concentrations were determined in samples diluted 1:20 using a sandwich ELISA kit (Cat. No: RKM100, R&D) according to the manufacturer's instructions. Urine KIM-1-to-creatinine ratios were calculated and presented in picogram of KIM-1 per millimole of creatinine (pg/mmol).

### Histological analyses

Kidney and heart were fixed with 4% paraformaldehyde for 24 h at room temperature, and then transferred to 70% ethanol. Tissues were embedded in paraffin blocks and sectioned at 5 µm. Following dewaxing and dehydration, kidney sections were stained with periodic acid-Schiff (PAS) for the quantification of tubular casts. For each kidney section, 10 pictures were randomly taken in both cortex and medulla at × 10 magnification, and the number of tubular casts was counted in each picture. Results are shown as total number of casts per kidney section.

CD68 immunodetection was used to identify macrophages and monocytes in the renal cortical interstitium of each rat. Following dewaxing and dehydration, antigen retrieval was performed by boiling kidney sections for 15 min in 10 mM citrate buffer (pH 6.0) using a pressure cooker. Endogenous peroxidase was blocked by incubating sections in 3% hydrogen peroxide solution (in 50% methanol) for 30 min. Sections were then blocked using serum-free protein block solution (Cat. No: X0909, Dako, Carpinteria, CA, USA) for 15 min, followed by 2.5% normal horse serum (Cat. No: MP-7402, Vector Laboratories, Newark, CA, USA) for 30 min. Sections were incubated overnight at 4 °C in mouse-anti-rat CD68, clone ED1 primary antibody (Cat. No: MCA341GA, Bio-Rad, Hercules, CA, USA) diluted in antibody diluent solution (Cat. No: 003118, Life Technologies, Frederick, MD, USA). Sections were incubated in ImmPRESS Horse Anti-Mouse IgG Polymer solution (Cat. No: MP-7402, Vector Laboratories, Newark, CA, USA) for 30 min. Sections were then incubated in 3,3′-diaminobenzidine (DAB) substrate solution (Vector Laboratories, Newark, CA, USA) for ~ 5 min, and counterstained with hematoxylin for ~ 10 s followed by Scott's tap water substitute for ~ 20 s. After gradual dehydration in ethanol and xylene, sections were mounted in Pertex mounting medium (CellPath, Newtown, UK). ~ 15 images from kidney cortex interstitium were taken with a 40 × objective. Images were analysed using the ImageJ software plugin Colour Deconvolution 2^22,23^ as follows: using set scale option, distances in pixels were converted to μm. Then, image colours were deconvoluted using the “H DAB” vector which automatically isolates the brown of DAB stain. Using the “adjust threshold” option and defining a pre-set threshold value, the area of brown stain was automatically covered in red pixels. The total area covered by these pixels was calculated and expressed in μm^2^. For each rat, data is presented as the mean DAB-positive area of 15 pictures.

For the semi-automated quantification of perivascular collagen area, kidney and heart sections were stained with picrosirius red (PSR, Cat. No: ab150681, Abcam, Cambridge, UK). Images of intrarenal and intracardiac vessels were taken with 10 × objective and were analysed using ImageJ software as follows: first, vessel area was measured manually by defining a region of interest (ROI). Then, image was converted to red/green/blue (RGB) stack and the green channel was selected as it best highlights the collagen fibres. Using the “adjust threshold” option, perivascular collagen area was manually coloured in red pixels. The total area covered by these pixels was calculated and normalized to vessel area.

All analyses were performed by the same individual blinded to experimental groups.

### Data analyses and statistics

Data are expressed as means ± standard deviation (SD). Data were analysed using GraphPad Prism 6.0 software (GraphPad, La Jolla, CA, USA). Unpaired t-test was used for data where only two groups were compared. For data where two variables were compared, two-way ANOVA was used followed by Holm–Sidak post-hoc analysis for multiple comparisons. For wire myography studies, concentration–response curves were fitted with the data using nonlinear regression analysis and groups were compared using two-way ANOVA. For all analyses, p < 0.05 was considered statistically significant.

## Results

### Confirmation and validation of the rat model

PCR amplified a 379 bp region within exon 2 of *P2rx7* gene and confirmed the two-adenine bp insertion mutation, predicted to generate a premature stop codon. A schematic of the targeting and an example of Sanger sequencing of the PCR products from a *P2rx7*^*−/−*^ and a WT rat is shown in Supplementary Fig. 1.

**Figure 2:**
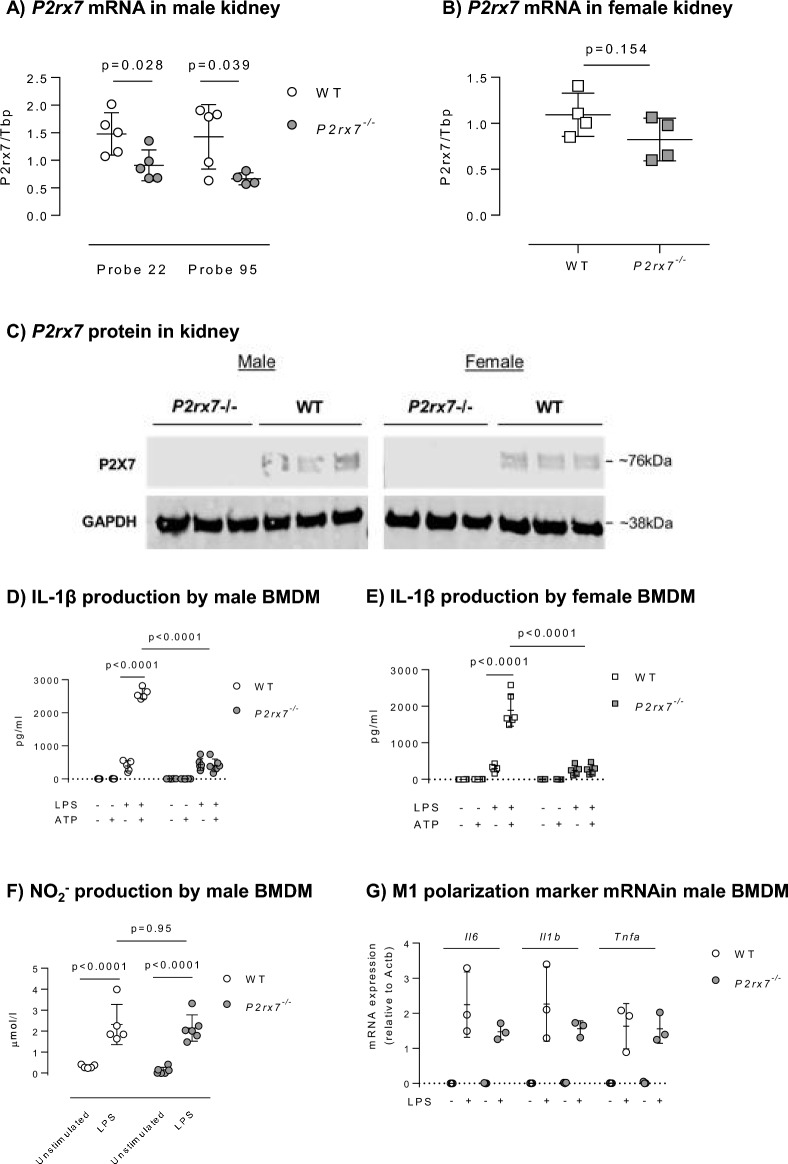
Validation of P2X7 deletion in *P2rx7*^*−/−*^ rats. (**A**) Partial decrease in *P2rx7* mRNA abundance in the kidney of male *P2rx7*^*−/−*^ vs. WT using two PCR primer sets (n = 5 rats/group). (**B**) Trend for a partial decrease in *P2rx7* mRNA abundance in the kidney of female *P2rx7*^*−/−*^ vs. WT (n = 4 rats/group). (**C**) Western blot demonstrating the absence of P2X7 protein expression in kidney samples of male and female *P2rx7*^*−/−*^ rats using an antibody directed against the C‐terminus of P2X7. GAPDH expression was used as a loading control (n = 3 rats/group). (**D**) Suppressed IL‐1β production by BMDM from male rats primed for 4 h with 1 μg/mL of LPS, then stimulated for 1 h with 3 mM ATP (n = 5–6 rats/group). (**E**) Suppressed IL‐1β production by BMDM from female rats primed for 4 h with 1 μg/mL of LPS, then stimulated for 1 h with 3 mM ATP (n = 5–6 rats/group). (**F**) Similar NO_2_^−^ production by male WT and *P2rx7*^*−/−*^ BMDM following stimulation with LPS for 4 h (n = 5–6 rats/group). (**G**) Similar induction of M1 polarisation marker genes by male WT and *P2rx7*^*−/−*^ BMDM following stimulation with LPS for 4 h (n = 3 rats/group). Data are means ± SD and statistical analysis performed using t-test (**A**,**B**) or 2-way ANOVA with Holm–Sidak post hoc correction (**D**–**G**). For all analyses, P < 0.05 was considered significant.

RT-qPCR was used to measure total kidney *P2rx7* mRNA abundance in age-matched adult male and female *P2rx7*^*-/-*^ and WT rats. We used four qPCR primer sets targeting different regions of *P2rx7* sequence (Supplementary Fig. 2A) to detect P2X7 mRNA expression in male kidneys, finding reduced expression in two primer sets (Fig. 2A; UPL probes 22 and 95) but not in the others (Supplementary Fig. 2B; UPL probes 12 and 65). Probe 95 was used to assess *P2rx7* abundance in female rat kidney and here there was no difference between genotypes (Fig. 2B).

We used the Alomone APR-004 antibody, which recognises an epitope corresponding to residues 576–595 of rat P2X7 receptor (exon 13), to screen bulk kidney protein extracts for P2X7 receptor protein. A band corresponding to P2X7 monomer was detected in WT rats but not in *P2rx7* knockouts of either sex (Fig. 2C). The full membrane images, including protein ladder are shown in Supplementary Fig. 3.

In LPS-primed BMDM, extracellular ATP activates P2X7 receptors to stimulate the release of IL-1β^24,25^. We confirmed ATP-stimulated IL-1β release in BMDM from male (Fig. 2D) and female WT rats (Fig. 2E). *P2rx7* mRNA abundance was reduced by ~ 60% (Supplementary Fig. 4) and ATP no longer evoked IL-1β release in *P2rx7*^*−/−*^, consistent with loss of a functional receptor. There was no sex difference in this phenotype. LPS-stimulation of nitric oxide production by BMDM was not impaired by P2X7 knockout (Fig. 2F). BMDM mRNA abundance for *Il6*, *Il1b* and *Tnf* was similar, indicating that loss of P2X7 receptors did not impair M1 polarisation of macrophages by LPS (Fig. 2G). BMDM mRNA abundance for *P2rx4* was not affected by P2X7 knockout, indicating that loss of P2X7 receptors was not compensated by increased abundance of a closely related receptor of the P2X family, at least in BMDM from male rats (Supplementary Fig. 4).

### Ex vivo vascular contractility

**Figure 3:**
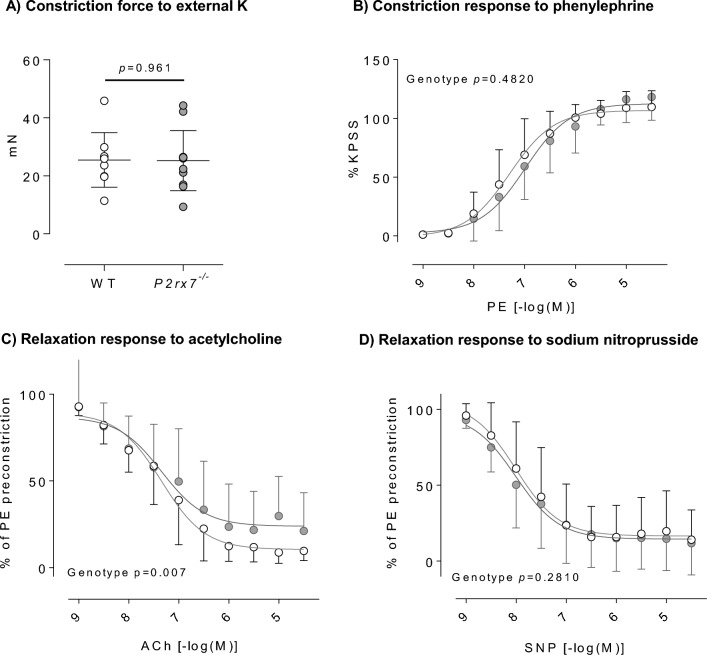
Ex vivo renal artery contractility in male *P2rx7*^*−/−*^ rats. (**A**) Similar external K^+^-evoked constriction force in male WT and *P2rx7*^*−/−*^ rat renal artery. (**B**) Similar vasoconstriction of male WT and *P2rx7*^*−/−*^ rat renal arteries to increasing phenylephrine (PE) concentrations. (**C**) Impaired vasodilation of male *P2rx7*^*−/−*^ rat renal arteries to increasing acetylcholine (ACh) concentrations. (**D**) Similar vasodilation of male WT and *P2rx7*^*−/−*^ rat renal arteries to increasing sodium nitroprusside (SNP) concentrations. For all, n = 9–11 rats/group. Data are means ± SD and statistical analysis performed using t-test (**A**) or 2-way ANOVA (**B**–**D**). For all analyses, P < 0.05 was considered significant.

Wire myography was used to measure ex vivo contractile responses of the renal artery. There were no genotype or sex difference in the contractile force generated in response to smooth muscle cell depolarisation induced by 125 mmol/L extracellular potassium (Fig. 2A and Supplementary Fig. 5A). In male rats, we found no difference in the response to phenylephrine (Fig. 3B) but the maximum relaxation induced by acetylcholine (endothelium-dependent) was significantly reduced in *P2rx7*^*−/−*^ rats (Fig. 3C). Endothelium-independent relaxation evoked by sodium nitroprusside (SNP) was similar between genotypes (Fig. 3D). SNP is a nitric oxide donor which provokes vasodilation by acting directly on the smooth muscle cells. The fact that the acetylcholine response was diminished, whereas the SNP response was not, suggests that P2X7 knockout reduces endothelial-derived nitric oxide bioavailability in the renal artery but does not affect sensitivity of the smooth muscle to nitric oxide.

In female rats, the contractile response to the alpha-adrenoceptor agonist, phenylephrine was not different between genotypes. Similarly, the relaxation induced by acetylcholine was not different between genotypes. The renal artery response to SNP was slightly enhanced by P2X7 receptor knockout (Supplementary Fig. 5).

We previously reported that AZ11657312, an antagonist of the rat P2X7 receptor, reduced renal artery resistance in vivo^13^. We therefore measured the contractile response of the renal artery to phenylephrine before and after a 15-min incubation with AZ11657312 (10 µM). In male wild-type rats AZ11657312 did not change the maximum vasoconstriction (Supplementary Fig. 6A) but induced a right-shift of the concentration–response curve in wild-type rats, so that sensitivity to phenylephrine was reduced (LogEC_50_ control = 6.64 ± 0.06 vs LogEC_50_ AZ11657312 = 5.94 ± 0.05; p < 0.0001). However, AZ11657312 caused a similar rightward displacement of the curve in male *P2rx7*^*−/−*^ rats (Supplementary Fig. 6B), significantly reducing the sensitivity of the renal artery to phenylephrine (LogEC_50_ control = 6.35 ± 0.07vs LogEC_50_ AZ11657312 = 5.86 ± 0.05; p < 0.0001), additionally reducing the maximal constriction (E_max_ control = 163.1 ± 4.6%, vs E_max_ AZ11657312 = 146.6 ± 4.0% vs. p = 0.014).

### In vivo renal hemodynamic and tubule function

**Figure 4:**
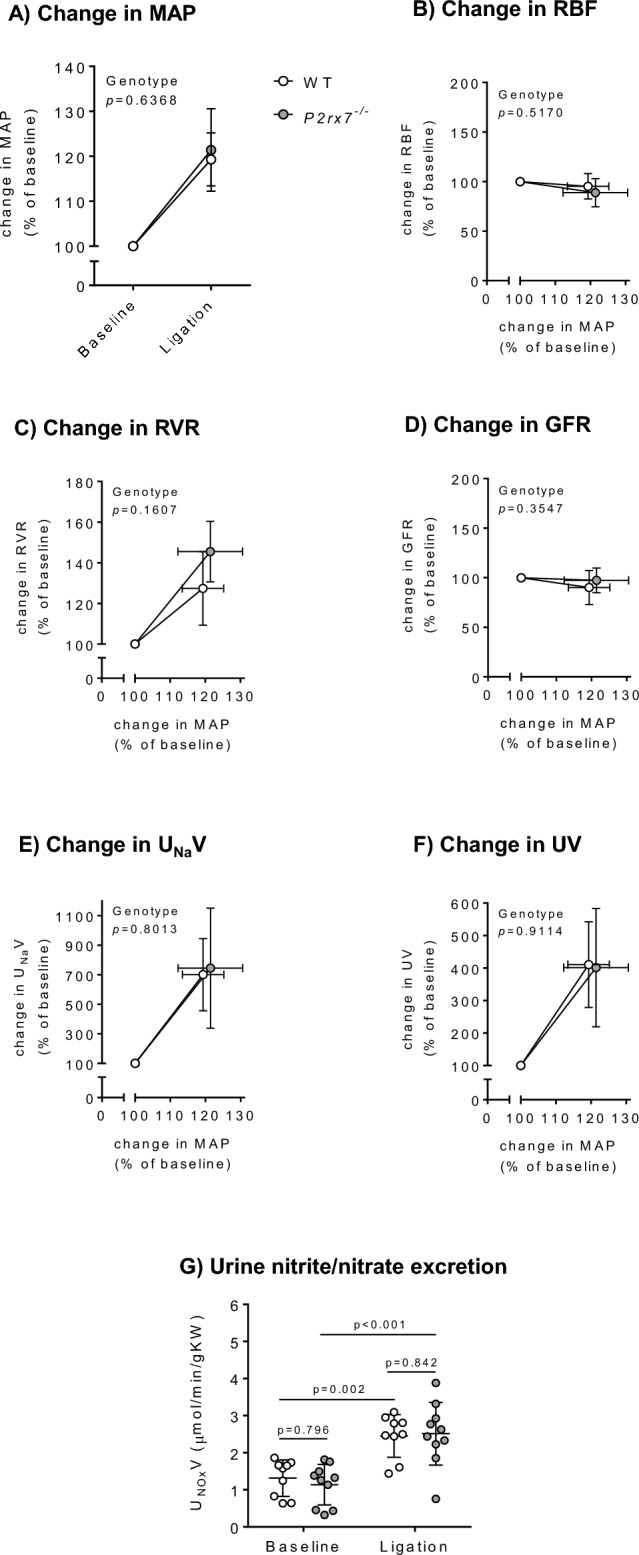
In vivo renal hemodynamics and the pressure natriuresis relationship in male *P2rx7*^*−/−*^ rats. (**A**) Change in mean arterial pressure (MAP) following ligation of coeliac, superior mesenteric, and distal aorta ligation. (**B**) Change in renal artery blood flow (RBF) as measured using a Transonic Doppler flow probe placed around the right main renal artery. (**C**) Change in renal vascular resistance (RVR). (**D**) Change in glomerular filtration rate (GFR). (**E**) Change in urinary sodium excretion rate (U_Na_V). (**F**) Change in urine flow rate (UV). (**G**) Urinary nitrite/nitrate excretion rate (U_NOx_V). For all, n = 9–10 rats/group. Data are means ± SD and statistical analysis performed using t-test (**A**–**F**) or 2-way ANOVA with Holm–Sidak post hoc correction (**G**). For all analyses, P < 0.05 was considered significant.

WT and *P2rx7*^−/−^ rats were anesthetised for measurement of RBF, GFR and electrolyte excretion. There were no differences in baseline parameters between genotypes in either male rats (Supplementary Table 2) or female (Supplementary Table 3). We then used arterial ligation to impose two successive pressure ramps and assessed renal hemodynamic and excretory responses to this intervention. In male rats, arterial ligations provoked a ~ 20% rise in mean arterial pressure (MAP) in both genotypes (Fig. 4A), while RBF remained stable (Fig. 4B) thus causing renal vascular resistance (RVR) to increase similarly in WT and *P2rx7*^*−/−*^ rats (Fig. 4C). GFR remained stable, consistent with renal autoregulatory capacity (Fig. 4D). The pressure natriuresis (Fig. 4E) and diuresis (Fig. 4F) responses were similar between WT and *P2rx7*^*-/-*^ rats. In female rats, arterial ligations provoked a 12–15% increase in MAP in both genotypes (Supplementary Fig. 7A). RBF remained stable (Supplementary Fig. 7B) and RVR increased similarly in both genotypes (Supplementary Fig. 7C). GFR remained stable in both genotypes (Supplementary Fig. 7D). Acute increases in renal perfusion pressure elicited the pressure natriuresis (Supplementary Fig. 7E) and diuresis (Supplementary Fig. 7F) responses, with no difference between WT and *P2rx7*^*−/−*^ rats. Overall, we found no difference between WT and *P2rx7*^*−/−*^ for any BP and kidney function parameters, including systolic BP (SBP), diastolic BP (DBP), heart rate, renal cortical and medullary blood perfusion, and urinary potassium and chloride excretion rate (Supplementary Figs. 8–10).

Given the renal vascular difference induced by P2X7 KO was only observed in male rats, the remaining experiments were conducted in male rats only. We quantified the urinary excretion rate of nitrite and nitrate (NOx) in male rats, as a surrogate measure of systemic and renal NO production. We found that P2X7 KO did not affect basal NO production and the elevation in NO production following acute increases in BP (Fig. 4G).

### Radio-telemetry blood pressure

**Figure 5:**
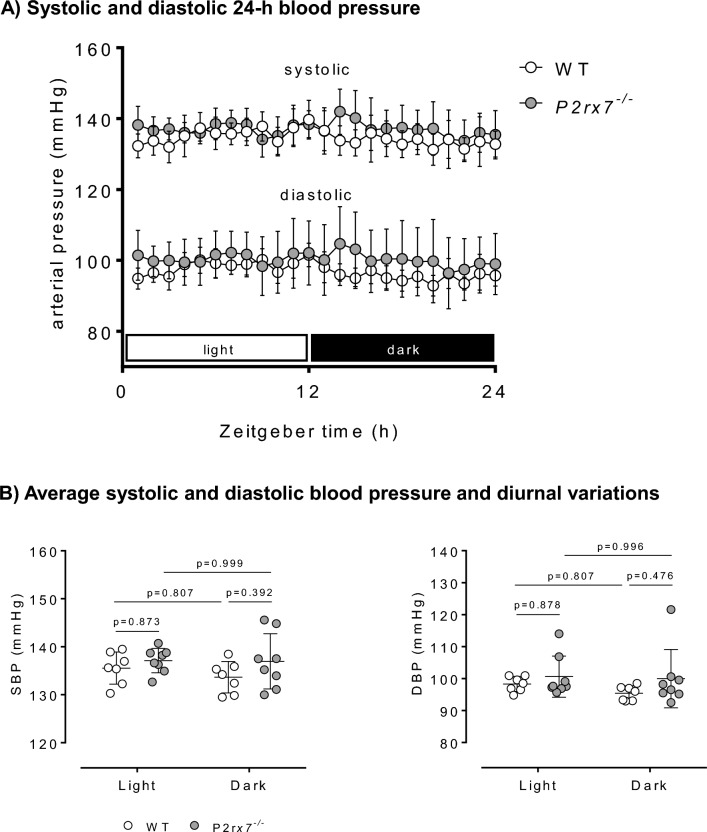
Radiotelemetry blood pressure in healthy male *P2rx7*^*−/−*^ rats. Blood pressure was recorded for 5 consecutive days using radiotelemetry devices. Data from each 24 h period were averaged. (**A**) 24 h systolic and diastolic blood pressure profiles in WT and *P2rx7*^*−/−*^ rats. (**B**) Average systolic and diastolic blood pressure during light (inactive) and dark (active) 12 h phases in WT and *P2rx7*^*−/−*^ rats. n = 7–8 rats/group. Data are means ± SD and statistical analysis performed using 2-way ANOVA with Holm–Sidak post hoc correction (**B**). For all analyses, P < 0.05 was considered significant.

SBP and DBP were measured in conscious, unrestrained male *P2rx7*^*−/−*^ and WT rats using radio-telemetry over a period of five consecutive days (Fig. 5A). In both WT and *P2rx7*^*−/−*^ rats, averaged SBP and DBP remained similar between light and dark phases (Fig. 5B), suggesting that BP rhythm does not follow a nocturnal dipping pattern in F344 rats, as previously reported elsewhere^26,27^. Overall, averaged SBP and DBP were similar between WT and *P2rx7*^*−/−*^ rats (Fig. 5B), suggesting that P2X7 knockout does not alter BP in F344 rats.

### Response to chronic ANGII infusion

**Figure 6:**
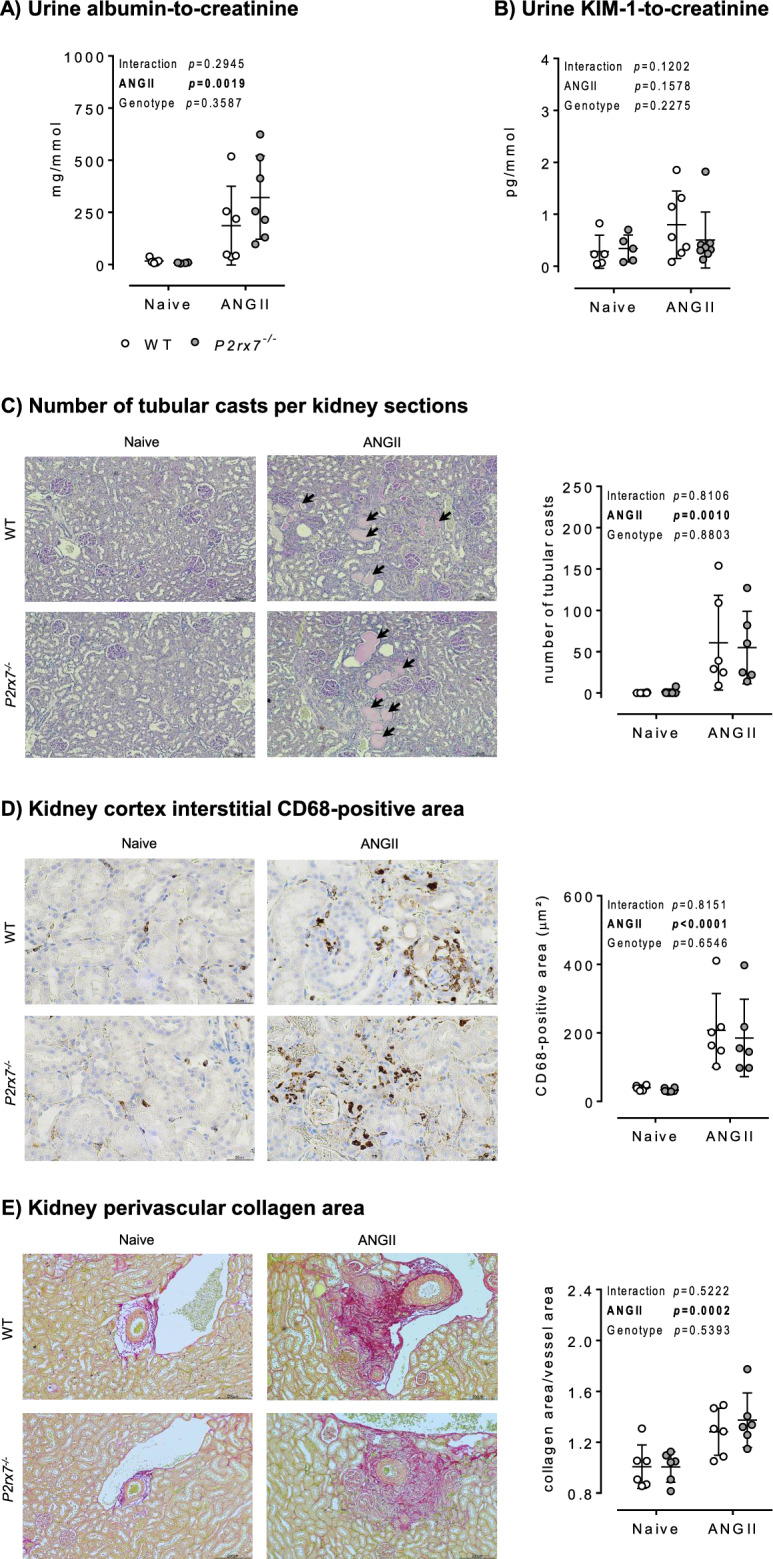
Kidney injury, inflammation, and fibrosis in male *P2rx7*^*−/−*^ rats following chronic ANGII infusion. Male WT and *P2rx7*^*−/−*^ rats underwent 5–6 week ANGII infusion. Urine and kidneys were harvested at end of infusion for the quantification of kidney injury, inflammation and fibrosis. (**A**) Quantification of albuminuria following ANGII infusion (n = 4–7 rats/group). (**B**) Quantification of the tubular injury biomarker KIM-1 in urine following ANGII infusion (n = 5–8 rats/group). (**C**) Number of tubular casts in kidney sections stained with PAS (n = 6 rats/group). (**D**) Quantification of the macrophage marker CD68-positive area in kidney sections (n = 6 rats/group). (**E**) Quantification of perivascular collagen area in kidney sections (n = 6 rats/group). Data are means ± SD and statistical analysis performed using 2-way ANOVA with Holm–Sidak post hoc correction to test for the effect of angiotensin II (ANGII), *P2rx7* knockout (Genotype), and the interaction. For all analyses, P < 0.05 was considered significant.

Male WT and *P2rx7*^*−/−*^ rats were infused ANGII for ~ 6 weeks. This induced significant albuminuria, with no difference between genotypes (Fig. 6A). Urinary levels of the tubular injury biomarker KIM-1, however, were not significantly increased by chronic ANGII-infusion in either P2X7 or WT rats (Fig. 6B).

In naïve rats, there was no difference in kidney weight normalized to tibia length between WT and *P2rx7*^*−/−*^ rats. Heart weight normalized to tibia length, however, was significantly lower in *P2rx7*^*−/−*^ rats compared to WT (Supplementary Table S4). ~ 6 weeks ANGII infusion provoked a decrease in kidney weight-to-tibia length ratio in both genotypes, but this was statistically significant in WT only (Supplementary Table S4). ANGII infusion significantly increased heart weight-to-tibia length ratio to similar values in both genotypes (Supplementary Table S4).

Histological analyses of glomeruli on PAS-stained kidney sections found evidence of occasional, focal, segmental hyalinosis, but most glomeruli were undamaged. In contrast, ANGII infusion caused tubulo-interstitial damage, particularly at the corticomedullary region. We observed frequent areas of tubular dilation and atrophy, and numerous tubular casts in both genotypes. We counted the total number of tubular casts per kidney section and found no significant difference between WT and *P2rx7*^*−/−*^ rats (Fig. 6C).

ANGII infusion was associated with focal mononuclear cells infiltration into the kidney. CD68 immunostaining was used to identify macrophages and monocytes in the renal cortex interstitium (Fig. 6D). In naïve rats, sparse single CD68-positive cells were detected across the cortical interstitium. ANGII infusion largely increased the area of CD68 staining, particularly in regions where tubular damage and perivascular fibrosis appeared more severe. Loss of P2X7 did not affect CD68 immunostaining in either naive or ANGII-infused rats (Fig. 6D).

Using PSR stain, we quantified the area of collagen deposition around blood vessels in the kidney (Fig. 6E) and myocardium (Supplementary Fig. 11). We found that ANGII infusion was associated with a significant increase in perivascular collagen area that was similar between WT and *P2rx7*^*−/−*^ rats, indicative of similar renal and cardiac perivascular fibrosis in this model.

Wire myography was used to measure ex vivo contractile responses of the renal artery (Supplementary Fig. 12). The contractile force generated in response to smooth muscle cell depolarisation was not altered by ANGII infusion in both genotypes. ANGII infusion did not significantly alter the relaxation induced by acetylcholine in both WT and *P2rx7*^*−/−*^ rats. Similarly, the response to sodium nitroprusside was not altered by ANGII in WT rats. In *P2rx7*^*−/−*^ rats, we found no difference in the maximum relaxation induced by sodium nitroprusside, however ANGII induced a right-shift of the concentration–response curve (LogIC_50_ naïve = 8.09 ± 0.16 vs LogIC_50_ ANGII = 7.27 ± 0.11; *p* = 0.0001).

## Discussion

Previous studies have indicated that P2X7 receptor activation promotes constriction of renal arteries and afferent arterioles^13,15,28^. Receptor blockade or knockout protects against kidney fibrosis and albuminuria in some experimental disease models^28–32^. We generated a P2X7 knockout rat to examine the contribution of the receptor in renal physiology and to the development of kidney injury caused by a ~ 6-week exposure to angiotensin II. We found a modest contribution of P2X7 receptor activation to nitric oxide-dependent vasodilation in the renal artery, assessed ex vivo. This was observed in male but not female rats. Receptor knockout did not affect in vivo renal hemodynamics and sodium transport and did not prevent angiotensin II-induced renal injury.

### P2X7 receptor in renal artery physiology

The P2X7 receptor is expressed in arterial endothelial cells in humans and rodents but its function is unclear. Millimolar concentrations of extracellular ATP are required for P2X7 receptor activation^33,34^ and circulating levels are typically ~ 1000 times lower than this^35–38^, although tissue injury can substantially increase local ATP concentration. Non-nucleotide moieties such as NAD may also contribute to P2X7 receptor activation^39^ and splice variation can result in functional variants with increased sensitivity to ATP^40^. In 2012, we reported that acute infusion of the P2X7 receptor antagonist Brilliant Blue G decreased BP in healthy male rats^14^. We interpreted this as evidence of tonic P2X7 receptor activation in the peripheral vasculature, suggesting that activation of the receptor was intrinsically pro-constrictive. Brilliant Blue G is a potent P2X7 receptor antagonist, with a nanomolar affinity^41^, but is poorly selective inhibiting P2X4 receptors, P2X5 receptors and potentially non-purinergic targets. In subsequent studies we used AZ11657312, a rodent analogue of a potent P2X7 receptor antagonist with no reported activity at other P2 receptors or reported off target effects^42^. This antagonist had little effect on BP and renal hemodynamics in control male F344 rats. In animals infused with angiotensin II for two weeks, AZ11657312 lowered BP and increased renal perfusion and oxygenation^13^. Other groups found that structurally different P2X7 receptor antagonists reduced afferent arteriolar resistance and increase single nephron glomerular filtration rate in rats treated with angiotensin II but not in control animal^15,43^. Overall, these studies suggested that P2X7 receptor activation enhances peripheral and/or renal vascular constriction in settings where vascular tone is already high. However, the current study points in the opposite direction. Here, P2X7 receptor knockout attenuated the response of the renal artery to acetylcholine. The relaxation induced by the nitric oxide donor, sodium nitroprusside, was unaffected by P2X7 receptor knockout. Overall, this finding suggests that activation of endothelial P2X7 receptors normally potentiates with nitric oxide production to promote vasodilation of the renal artery. It is not clear how such an interaction might occur. We can speculate that this might relate to the intracellular calcium influx via P2X7 receptors. Additionally, P2X7 receptor knockout depletes cellular glutathione^44^, which would be anticipated to reduce endothelial cell nitric oxide bioavailability^45^.

The vascular phenotype observed in male P2X7 receptor rats did not translate to abnormalities in blood pressure or renal hemodynamic physiology and we found no evidence of reduced global nitric oxide bioavailability. Circulating NOx are freely filtered by the glomeruli and there is extensive reabsorption in the proximal tubule^46–48^ and it is possible that a small decrease in systemic nitric oxide production could have been masked. Nevertheless, our data using a genetic knockout strategy show that P2X7 receptors are not major regulators of cardiovascular and renal physiology, even in settings of elevated vascular tone. This contrasts with earlier studies. Life-long gene deletion offers a different context from acute pharmacological blockade and other pathways may compensate for P2X7 receptor knockout. For example, P2X7 interacts with the closely related P2X4 receptor and can form functional heteromeric receptors^49,50^. P2X4 receptors are highly expressed in human and rodent endothelial cells^17^ and may offer redundancy when P2X7 is deleted^51^. Off target effects of P2X7 receptor antagonists should also be considered since in the current study we found that AZ11657312 reduced sensitivity to phenylephrine in renal artery of the P2X7 receptor knockout rat. AZ11657312 is an adamantane-based compound identified through a high-throughput screening for novel P2X7 antagonists^42,52^. Hit-to-lead development took a small series of compounds with potent (nanomolar) inhibitory activity at human and rat P2X7 receptors and assessed their ability to antagonise BzATP-induced plasma membrane pore formation and interleukin-1β release. AZ11657312 selectively targeted the rat P2X7 receptor in these cell-based assays. It was additionally screened for activity against a panel of 153 enzyme and receptor assays. No inhibitory action against P2X1-5 was identified but affinity for the vasopressin V1A receptor, (IC_50_ 7 μM), and a nonselective sigma receptor (IC_50_ 4 μM) were reported^42^. It is possible that AZ11657312, used at 10 μM in our myography experiment, engaged V1A receptors in the vascular smooth muscle cells. Moreover, by current standards, the original screen is restricted in scope and the potential for other off-target effects cannot be excluded.

### Sex differences

Attenuated acetylcholine induced relaxation of the renal artery was not observed in female P2X7 receptor knockout mice. The renal vasculature of male rats is more dependent on nitric oxide production than that of females^53^. A small number of other studies have also shown sexual dimorphism in P2X7 receptor mediated responses, albeit in other contexts. For example, P2X7R knockout alters exocrine secretion by the salivary and lacrimal glands in male mice but not in female mice: the receptor is expressed in both sexes but in female mice exocrine secretion is less dependent on functional P2X7 receptors^54^. Knockout of the receptor significantly reduces bone mineral content and total bone mass by impairing periosteal bone formation. The effect is much more pronounced in male mice than females^55^. Other studies show qualitatively similar outcomes with pharmacological blockade of the receptor: Brilliant Blue G ameliorates neurodegeneration in a mouse model of ALS to a greater extent in males than in females^56^. Oestrogens do not influence P2X7 receptor expression, at least at the mRNA level^57^. However, 17β-oestradiol rapidly and reversibly reduces calcium flux through the P2X7 receptor and functionality of the P2X7 receptor may be non-genomically supressed in female mice^58,59^.

### Contribution of P2X7 receptor to the development of renal injury

Extracellular ATP is released from injured cells and P2X7 receptor activation leads to NLRP3 inflammasome activation, cleavage and release of IL-1β from immune cells^60^. Our study, and several others^25,61,62^, find that P2X7 receptor knockout abolishes ATP-stimulated production of IL-1β in LPS-primed BMDM. Unsurprisingly, P2X7 receptors have received much therapeutic interest in the context of inflammatory diseases and prophylactic antagonism is beneficial in experimental kidney and cardiovascular disease^6,63^. Chronic angiotensin II infusion is widely used in this context. In the rat, prophylactic treatment with Brilliant Blue G reduces the immune cell infiltration, inflammasome activation and accumulation of proinflammatory cytokines within kidney tissue caused by a 2-week exposure to angiotensin II^28^. In the mouse, pharmacological blockade and P2X7 receptor knockout attenuate hypertension, reducing perivascular accumulation of T-cells and endothelial dysfunction^61^. Surprisingly, given the beneficial effect on BP, P2X7 receptor blockade did not improve heart function or prevent the cardiac remodelling induced by angiotensin II and P2X7 receptor knockout aggravated this phenotype. In our study we infused angiotensin II for ~ 6 weeks, inducing cardiac hypertrophy, albuminuria, CD68 + monocyte/macrophage infiltration and perivascular fibrosis. We identified an equivalent level of injury/remodelling in wild-type and P2X7 receptor knockout rats. Similar findings have been reported for experimental glomerular nephritis: receptor antagonism and knockout are protective in mice^16^ whereas P2X7 receptor knockout rats show a similar level of kidney inflammation and injury^62^ and any pharmacological benefit of P2X7 receptor antagonists was due to off target effects. Species differences may also contribute importantly to P2X7 receptor biology and to the functional impact of genetic receptor modification or pharmacological inhibition. Indeed, in expression systems, mouse, rat and human P2X7 receptors display different pharmacological responses to several P2X7 agonists/antagonists, suggesting that the nature and function of P2X7 receptors may differ considerably between species^64^. This is in line with the previously reported species-specific variations in P2X7 receptor isoforms and splice variants which may account for differences in receptor function and signalling^34,40,65,66^. Given this complexity, it is perhaps not surprising that the precise contribution of P2X7 receptors to renal disease pathogenesis remains unclear. In the mouse, for example, antagonism and genetic deletion often provide protection against kidney disease; in the rat such approaches have little benefit and the receptor may be less important to disease development and progression. The lack of consensus from preclinical modelling presents a significant translational roadblock.

## Conclusion

Our study does not support a major role for P2X7 receptor activation in blood pressure and renal hemodynamic physiology in the rat. P2X7 receptor knockout prevented ATP-stimulated release of IL-1β from macrophages. IL-1β contributes to the injurious response of chronic angiotensin II infusion^67^ but our data indicate that P2X7 receptor activation is not required for the development of injury/fibrosis in the rat. Indeed, rats^62^ and humans^68^ have alternative pathways for IL-1β cleavage in monocytes, which may be why P2X7 receptor antagonists have not yet delivered benefit against chronic inflammatory outcomes in clinical trials^69^.

### Supplementary Information

Supplementary Information.

## Data Availability

The datasets generated and analysed during the current study are available in The University of Edinburgh’s DataShare repository (https://datashare.ed.ac.uk/handle/10283/929), with this persistent identifier: 10.7488/ds/7721.

## References

[1] Burnstock, G. Purines and purinoceptors: Molecular biology overview☆. In *Reference Module in Biomedical Sciences* B9780128012383047413 (Elsevier, 2014). 10.1016/B978-0-12-801238-3.04741-3.

[2] Burnstock G (2018). Purine and purinergic receptors. Brain Neurosci. Adv..

[3] Burnstock G, Evans LC, Bailey MA (2014). Purinergic signalling in the kidney in health and disease. Purinergic Signal..

[4] Vallon V, Unwin RJ, Inscho EW, Leipziger J, Kishore BK (2019). Extracellular nucleotides and P2 receptors in renal function. Physiol. Rev..

[5] Menzies RI, Unwin RJ, Bailey MA (2015). Renal P2 receptors and hypertension. Acta Physiol..

[6] Menzies RI, Tam FW, Unwin RJ, Bailey MA (2017). Purinergic signaling in kidney disease. Kidney Int..

[7] Grahames CB, Michel AD, Chessell IP, Humphrey PP (1999). Pharmacological characterization of ATP- and LPS-induced IL-1beta release in human monocytes. Br. J. Pharmacol..

[8] Mariathasan S (2006). Cryopyrin activates the inflammasome in response to toxins and ATP. Nature.

[9] Granata S (2015). NLRP3 inflammasome activation in dialyzed chronic kidney disease patients. PLoS One.

[10] Turner CM (2007). Increased expression of the pro-apoptotic ATP-sensitive P2X7 receptor in experimental and human glomerulonephritis. Nephrol. Dial. Transplant..

[11] Menzies RI (2017). Hyperglycemia-induced renal P2X7 receptor activation enhances diabetes-related injury. EBioMedicine.

[12] Zhu Y (2022). P2X7 receptor inhibition attenuates podocyte injury by oxLDL through deregulating CXCL16. Cell Biol. Int..

[13] Menzies RI (2015). Inhibition of the purinergic P2X7 receptor improves renal perfusion in angiotensin-II-infused rats. Kidney Int..

[14] Menzies, R. I. *et al.* Effect of P2X4 and P2X7 receptor antagonism on the pressure diuresis relationship in rats. *Front. Physiol.* **4**, (2013). 10.3389/fphys.2013.00305 PMC3807716 24187541

[15] Franco M (2017). Physiopathological implications of P2X_1_ and P2X_7_ receptors in regulation of glomerular hemodynamics in angiotensin II-induced hypertension. Am. J. Physiol.-Ren. Physiol..

[16] Taylor SRJ (2009). P2X7 deficiency attenuates renal injury in experimental glomerulonephritis. J. Am. Soc. Nephrol. JASN.

[17] Yamamoto K (2000). P2X4 receptors mediate ATP-induced calcium influx in human vascular endothelial cells. Am. J. Physiol. Heart Circ. Physiol..

[18] Crawford C (2011). Extracellular nucleotides affect pericyte-mediated regulation of rat in situ vasa recta diameter: Nucleotides affect pericyte-mediated regulation of vasa recta diameter. Acta Physiol..

[19] Culshaw GJ (2019). Impaired pressure natriuresis and non-dipping blood pressure in rats with early type 1 diabetes mellitus. J. Physiol..

[20] Roman RJ, Cowley AW, Garcia-Estañ J, Lombard JH (1988). Pressure-diuresis in volume-expanded rats. Cortical and medullary hemodynamics. Hypertens. Dallas Tex. 1979.

[21] Börner U, Szász G, Bablok W, Busch EW (1979). A specific fully enzymatic method for creatinine: Reference values in serum (author’s transl). J. Clin. Chem. Clin. Biochem. Z. Klin. Chem. Klin. Biochem..

[22] Ruifrok AC, Johnston DA (2001). Quantification of histochemical staining by color deconvolution. Anal. Quant. Cytol. Histol..

[23] Landini G, Martinelli G, Piccinini F (2021). Colour deconvolution: Stain unmixing in histological imaging. Bioinform. Oxf. Engl..

[24] Alarcón-Vila C (2020). CD14 release induced by P2X7 receptor restricts inflammation and increases survival during sepsis. eLife.

[25] Ryoden Y, Fujii T, Segawa K, Nagata S (2020). Functional expression of the P2X7 ATP receptor requires eros. J. Immunol..

[26] Basset A, Laude D, Laurent S, Elghozi J-L (2004). Contrasting circadian rhythms of blood pressure among inbred rat strains: Recognition of dipper and non-dipper patterns. J. Hypertens..

[27] Mullins, L. J., Koutraki, Y. G. S., Bailey, M. A. & Mullins, J. J. *Vagal Involvement in Non-Dipping Phenotype of Hsd11b2 Knockout Rats*. (2022) 10.1101/2022.02.11.480066.

[28] Bautista-Pérez R (2020). The role of P2X7 purinergic receptors in the renal inflammation associated with angiotensin II-induced hypertension. Int. J. Mol. Sci..

[29] Gonçalves RG (2006). The role of purinergic P2X7 receptors in the inflammation and fibrosis of unilateral ureteral obstruction in mice. Kidney Int..

[30] Ji X (2012). P2X7 receptor antagonism attenuates the hypertension and renal injury in Dahl salt-sensitive rats. Hypertens. Res..

[31] Ji X (2012). P2X _7_ deficiency attenuates hypertension and renal injury in deoxycorticosterone acetate-salt hypertension. Am. J. Physiol.-Ren. Physiol..

[32] Pereira, J. M. S. *et al.* Brilliant blue G, a P2X7 receptor antagonist, attenuates early phase of renal inflammation, interstitial fibrosis and is associated with renal cell proliferation in ureteral obstruction in rats. *BMC Nephrol.* **21**, (2020). 10.1186/s12882-020-01861-2 PMC7260756 32471386

[33] Surprenant A, Rassendren F, Kawashima E, North RA, Buell G (1996). The cytolytic P2Z receptor for extracellular ATP identified as a P2X receptor (P2X7). Science.

[34] Kopp, R., Krautloher, A., Ramírez-Fernández, A. & Nicke, A. P2X7 interactions and signaling—Making head or tail of it. *Front. Mol. Neurosci.* **12**, (2019). 10.3389/fnmol.2019.00183 PMC6693442 31440138

[35] Velasquez S (2020). Circulating levels of ATP is a biomarker of HIV cognitive impairment. EBioMedicine.

[36] Heinle H (1987). Metabolite concentration gradients in the arterial wall of experimental atherosclerosis. Exp. Mol. Pathol..

[37] Ryan LM, Rachow JW, McCarty BA, McCarty DJ (1996). Adenosine triphosphate levels in human plasma. J. Rheumatol..

[38] Buchet R (2021). Hydrolysis of extracellular ATP by vascular smooth muscle cells transdifferentiated into chondrocytes generates Pi but not PPi. Int. J. Mol. Sci..

[39] Di Virgilio, F., Giuliani, A. L., Vultaggio-Poma, V., Falzoni, S. & Sarti, A. C. Non-nucleotide agonists triggering P2X7 receptor activation and pore formation. *Front. Pharmacol.* **9**, (2018). 10.3389/fphar.2018.00039 PMC5799242 29449813

[40] Nicke A (2009). A functional P2X7 splice variant with an alternative transmembrane domain 1 escapes gene inactivation in P2X7 knock-out mice. J. Biol. Chem..

[41] Jiang LH, Mackenzie AB, North RA, Surprenant A (2000). Brilliant blue G selectively blocks ATP-gated rat P2X(7) receptors. Mol. Pharmacol..

[42] Furber M (2007). Discovery of potent and selective adamantane-based small-molecule P2X(7) receptor antagonists/interleukin-1beta inhibitors. J. Med. Chem..

[43] Kulthinee S, Shao W, Franco M, Navar LG (2020). Purinergic P2X1, P2X7 and angiotensin AT1 receptors interactions in the regulation of renal afferent arteriole in angiotensin II-dependent hypertension. Am. J. Physiol.-Ren. Physiol..

[44] Park H, Kim J-E (2020). Deletion of P2X7 receptor decreases basal glutathione level by changing glutamate-glutamine cycle and neutral amino acid transporters. Cells.

[45] Prasad A, Andrews NP, Padder FA, Husain M, Quyyumi AA (1999). Glutathione reverses endothelial dysfunction and improves nitric oxide bioavailability. J. Am. Coll. Cardiol..

[46] Sütő T (1995). Acute changes in urinary excretion of nitrite + nitrate do not necessarily predict renal vascular NO production. Kidney Int..

[47] Godfrey M, Majid DSA (1998). Renal handling of circulating nitrates in anesthetized dogs. Am. J. Physiol.-Ren. Physiol..

[48] Sundqvist, M. L., Lundberg, J. O., Weitzberg, E. & Carlström, M. Renal handling of nitrate in women and men with elevated blood pressure. *Acta Physiol.* **232**, (2021). 10.1111/apha.13637 33630408

[49] Guo C, Masin M, Qureshi OS, Murrell-Lagnado RD (2007). Evidence for functional P2X4/P2X7 heteromeric receptors. Mol. Pharmacol..

[50] Schneider M (2017). Interaction of purinergic P2X4 and P2X7 receptor subunits. Front. Pharmacol..

[51] Pochet S (2007). Contribution of two ionotropic purinergic receptors to ATP responses in submandibular gland ductal cells. Cell. Signal..

[52] Baxter A (2003). Hit-to-Lead studies: the discovery of potent adamantane amide P2X7 receptor antagonists. Bioorg. Med. Chem. Lett..

[53] Reckelhoff J (1998). Gender differences in the renal nitric oxide (NO) system dissociation between expression of endothelial NO synthase and renal hemodynamic response to NO synthase inhibition. Am. J. Hypertens..

[54] Novak I, Jans IM, Wohlfahrt L (2010). Effect of P2X(7) receptor knockout on exocrine secretion of pancreas, salivary glands and lacrimal glands. J. Physiol..

[55] Ke HZ (2003). Deletion of the P2X _7_ Nucleotide receptor reveals its regulatory roles in bone formation and resorption. Mol. Endocrinol..

[56] Cervetto C, Frattaroli D, Maura G, Marcoli M (2013). Motor neuron dysfunction in a mouse model of ALS: gender-dependent effect of P2X7 antagonism. Toxicology.

[57] Barabási B (2016). Effect of axotomy and 17β-estradiol on P2X7 receptor expression pattern in the hypoglossal nucleus of ovariectomized mice. Neuroscience.

[58] Cario-Toumaniantz C, Loirand G, Ferrier L, Pacaud P (1998). Non-genomic inhibition of human P2X _7_ purinoceptor by 17β-oestradiol. J. Physiol..

[59] Gorodeski GI (2004). Estrogen attenuates P2X7-R-mediated apoptosis of uterine cervical cells by blocking calcium influx. Nucleosides Nucleotides Nucleic Acids.

[60] Ferrari D (2006). The P2X _7_ receptor: A key player in IL-1 processing and release. J. Immunol..

[61] Shokoples BG (2023). P2RX7 gene knockout or antagonism reduces angiotensin II-induced hypertension, vascular injury and immune cell activation. J. Hypertens..

[62] Prendecki M (2022). Glomerulonephritis and autoimmune vasculitis are independent of P2RX7 but may depend on alternative inflammasome pathways. J. Pathol..

[63] Shokoples BG, Paradis P, Schiffrin EL (2020). P2X7: An untapped target for the management of cardiovascular disease. Arterioscler. Thromb. Vasc. Biol..

[64] Donnelly-Roberts DL, Namovic MT, Han P, Jarvis MF (2009). Mammalian P2X7 receptor pharmacology: Comparison of recombinant mouse, rat and human P2X7 receptors. Br. J. Pharmacol..

[65] Benzaquen J (2019). Alternative splicing of P2RX7 pre-messenger RNA in health and diseases: Myth or reality?. Biomed. J..

[66] De Salis SKF (2022). Alternatively spliced isoforms of the P2X7 receptor: Structure, function and disease associations. Int. J. Mol. Sci..

[67] Akita K (2021). Blocking of interleukin-1 suppresses angiotensin II-induced renal injury. Clin. Sci. Lond. Engl..

[68] Gaidt MM (2016). Human monocytes engage an alternative inflammasome pathway. Immunity.

[69] Pelegrin P (2021). P2X7 receptor and the NLRP3 inflammasome: Partners in crime. Biochem. Pharmacol..

