# Supplementary material for: P2X7 receptor knockout does not alter renal function or prevent angiotensin II-induced kidney injury in F344 rats
Source: Sci Rep. 2024 Apr 26;14:9573. doi: 10.1038/s41598-024-59635-x (PMC11053004; doi:10.1038/s41598-024-59635-x)
Supplement: Supplementary file 1 — Supplementary Information. [file 41598_2024_59635_MOESM1_ESM.docx]

**Online data supplement**

**P2X7 Receptor Knockout Does Not Alter Renal Function or Prevent Angiotensin II-Induced Kidney Injury in F344 Rats**

Josselin Nespoux, Marie-Louise T Monaghan, Natalie K Jones, Kevin Stewart, Laura Denby, Alicja Czopek, John J Mullins, Robert I Menzies, Andrew H Baker, Matthew A Bailey

Edinburgh Kidney, British Heart Foundation Centre for Cardiovascular Science, The University of Edinburgh, United Kingdom

For Correspondence: Matthew Bailey, PhD, FRSB

[Matthew.bailey@ed.ac.uk](mailto:Matthew.bailey@ed.ac.uk)

**Word count:**

**Figures:** 5 main; 12 supplemental

**Tables:** 4 supplemental

**Short title:** Renal injury in P2X7 knockout rats

**Supplemental Table 1: Real-time qPCR primers used**

Genes identities and corresponding forward and reverse primer sequences, with the specific UPL probe number and the length of the qPCR products. 4 different primer sets were used to detect *P2rx7* mRNA. *Actb*, beta-actin; *Il1b*, interleukin-1β; *Il6*, interleukin-6; *P2rx4*, purinergic receptor P2X4; *P2rx7*, purinergic receptor P2X7; *Tbp*, TATA-box binding protein; *Tnf,* tumor necrosis factor alpha.

| **Gene** | **Forward** | **Reverse** | **Probe number** | **Amplicon size (nt)** |
| --- | --- | --- | --- | --- |
| *Actb* | cccgcgagtacaaccttct | cgtcatccatggcgaact | 17 | 72 |
| *Il1b* | tgtgatgaaagacggcacac | cttcttctttgggtattgtttgg | 78 | 70 |
| *Il6* | cctggagtttgtgaagaacaact | ggaagttggggtaggaagga | 106 | 142 |
| *P2rx4* | gcctgcccagatattcctt | ccctgtagtacttggcaaacct | 130 | 96 |
| *P2rx7* | gcacatgaccgtcttttcct | ttggtgtgcacagagctgat | 12 | 93 |
|  | ctgcaagatgtcaaaggtcaag | tcaggttgtccaggaattgg | 22 | 110 |
|  | ttttgacatcctggtttttgg | tggatccaatgtacacaacca | 65 | 71 |
|  | ttacagaggtggcagttcagg | agctgtatttgggttgacagc | 95 | 99 |
| *Tbp* | cccaccagcagttcagtagc | caattctgggtttgatcattctg | 129 | 75 |
| *Tnf* | tgaacttcggggtgatcg | gggcttgtcactcgagtttt | 63 | 122 |

**Supplemental Table 2: Baseline parameters in anesthetized male F344 WT and *P2rx7^-/-^* rats**

Data are means ± SD and statistical analysis performed using t-test; n=9-10 rats per genotype. For all analyses, P<0.05 was considered significant. BW, body weight; MAP, mean arterial blood pressure; SBP, systolic blood pressure; DBP, diastolic blood pressure; HR, heart rate; BPU, blood perfusion unit; LDF, laser doppler flow; RBF, renal blood flow; RVR, renal vascular resistance; GFR, glomerular filtration rate; UV, urine flow rate; U_Na_V, urinary sodium excretion rate; U_K_V, urinary potassium excretion rate; U_Cl_V, urinary chloride excretion rate; U_NOx_V, urinary nitrite/nitrate excretion rate.

| **Parameters** | **WT** | ***P2rx7^-/-^*** | **P value** |
| --- | --- | --- | --- |
| BW (g) | 382±17 | 333±9 | <0.0001 |
| MAP (mmHg) | 156±13 | 149±12 | 0.2725 |
| SBP (mmHg) | 171±18 | 161±13 | 0.2193 |
| DBP (mmHg) | 142±11 | 136±13 | 0.3455 |
| HR (bpm) | 422±28 | 413±22 | 0.4763 |
| Cortical perfusion (BPU) | 1559±388 | 1628±256 | 0.6671 |
| Medullary perfusion (LDF) | 549±225 | 606±300 | 0.6694 |
| RBF (mL/min) | 6.9±1.3 | 6.0±2.0 | 0.4531 |
| RVR (mmHg/mL.min^-1^) | 24±5 | 25±7 | 0.7048 |
| GFR (mL/min/gKW) | 0.94±0.14 | 0.88±0.16 | 0.4286 |
| UV (µL/min/gKW) | 6.2±1.6 | 5.1±1.5 | 0.1790 |
| U_Na_V (µmol/min/gKW) | 1.86±0.79 | 1.16±0.56 | 0.0549 |
| U_K_V (µmol/min/gKW) | 2.97±0.38 | 2.52±0.82 | 0.1777 |
| U_Cl_V (µmol/min/gKW) | 2.21±0.63 | 2.20±0.59 | 0.9722 |
| U_NOx_V (µmol/min/gKW) | 1.31±0.46 | 1.14±0.52 | 0.4754 |

**Supplemental Table 3: Baseline parameters in anesthetized female F344 WT and *P2rx7^-/-^* rats**

Data are means ± SD and statistical analysis performed using t-test; n=9-10 rats per genotype. For all analyses, P<0.05 was considered significant. BW, body weight; MAP, mean arterial blood pressure; SBP, systolic blood pressure; DBP, diastolic blood pressure; HR, heart rate; BPU, blood perfusion unit; LDF, laser doppler flow; RBF, renal blood flow; RVR, renal vascular resistance; GFR, glomerular filtration rate; UV, urine flow rate; U_Na_V, urinary sodium excretion rate; U_K_V, urinary potassium excretion rate; U_Cl_V, urinary chloride excretion rate; U_NOx_V, urinary nitrite/nitrate excretion rate.

| **Parameters** | **WT** | ***P2rx7^-/-^*** | **P value** |
| --- | --- | --- | --- |
| BW (g) | 208±7 | 197±9 | 0.0110 |
| MAP (mmHg) | 150±7 | 146±17 | 0.4744 |
| SBP (mmHg) | 158±9 | 154±18 | 0.5606 |
| DBP (mmHg) | 141±7 | 137±17 | 0.5257 |
| HR (bpm) | 406±29 | 425±22 | 0.4461 |
| Cortical perfusion (BPU) | 1332±328 | 1312±350 | 0.9023 |
| Medullary perfusion (LDF) | 479±236 | 634±406 | 0.3562 |
| RBF (mL/min) | 3.8±1.2 | 2.7±1.3 | 0.2442 |
| RVR (mmHg/mL.min^-1^) | 43±10 | 63±28 | 0.2155 |
| GFR (mL/min/gKW) | 0.86±0.25 | 0.90±0.24 | 0.7562 |
| UV (µL/min/gKW) | 5.1±1.5 | 4.5±1.9 | 0.4838 |
| U_Na_V (µmol/min/gKW) | 0.94±0.49 | 0.75±0.56 | 0.4864 |
| U_K_V (µmol/min/gKW) | 3.17±0.68 | 2.96±0.74 | 0.5477 |
| U_Cl_V (µmol/min/gKW) | 2.28±0.67 | 2.02±1.03 | 0.5534 |
| U_NOx_V (µmol/min/gKW) | 1.29±0.64 | 1.27±0.87 | 0.9624 |

**Supplemental Table 4: Body parameters in response to chronic ANGII infusion in male F344 WT and *P2rx7^-/-^* rats**

Data are means ± SD and statistical analysis performed using 2-way ANOVA with Holm-Sidak post hoc correction. # P<0.05 vs. naive rats; * P<0.05 vs. WT rats. Corresponding P values indicated in parentheses. For all, n=8-11/group.

|  | **WT naive** | ***P2rx7^-/-^* naive** | **WT ANGII** | ***P2rx7^-/-^* ANGII** |
| --- | --- | --- | --- | --- |
| Age on cull day (months) | 4.5 | 4 | 4.5 | 5.5 |
| BW (g) | 382±17 | 333±9 * (*p*<0.0001) | 298±27 # (*p*<0.0001) | 317±20 |
| Tibia length (mm) | 53±0.9 | 53±0.5 | 53±2.0 | 55±1.0 # (*p*<0.01) |
| Heart weight (g) | 1.00±0.08 | 0.89±0.05 * (*p*<0.01) | 1.10±0.08 # (*p*<0.01) | 1.11±0.05 # (*p*<0.0001) |
| Total kidney weight (g) | 2.24±0.04 | 2.09±0.04 | 1.92±0.07 # (*p*<0.001) | 2.02±0.17 |
| Heart weight/tibia length (mg/mm) | 18.6±1.4 | 16.9±0.8 * (*p*<0.01) | 20.5±0.9 # (*p*<0.01) | 20.3±0.8 # (*p*<0.0001) |
| Total kidney weight/tibia length (mg/mm) | 41.9±2.3 | 39.7±2.2 | 35.9±2.4 # (*p*<0.001) | 37.0±3.0 |

**Supplemental Figure 1: Generation of a novel *P2rx7^-/-^* rat**

A) Schema of CRISPR-induced insertion in exon 2 of the rat *P2rx7* gene. B) Schema of premature “STOP” codon (star symbol) in P2X7 protein resulting from the frameshift mutation. C) Example of Sanger sequencing of WT (top) and *P2rx7^-/-^* (bottom) rat showing a 2-adenine insertion (arrow) in *P2rx7^-/-^* sequence.

**Supplemental Figure 2: Detection of *P2rx7* mRNA in kidney**

A) Schema of qPCR primers targeting sites for the detection of *P2rx7* mRNA. B) Kidney mRNA abundance for *P2rx7* in male rats using primer pairs employing UPL probes 12 and 65 (n=5 rats/genotype). Data are means ± SD and statistical analysis performed using t-test. P<0.05 was considered significant.

**Supplemental Figure 3: Full membrane images of Western blot for rat P2X7 in kidney lysates**

Protein was extracted from kidney lysates from male and female WT and *P2rx7^-/-^* rats (n=3 rats/group). P2X7 protein was detected using Alomone APR-004 antibody. GAPDH was used as a loading control. Arrows indicate bands corresponding to the expected molecular weight for P2X7 monomer and for GAPDH.

**Supplemental Figure 4: *P2rx7* and *P2rx4* mRNA detection in BMDM**

A) BMDM mRNA abundance for *P2rx7* in male rats using primer pair employing UPL probes 95 (n=3 rats/genotype). B) BMDM mRNA abundance for *P2rx4* in male rats using primer pair employing UPL probes 95 (n=3 rats/genotype). Data are means ± SD and statistical analysis performed using 2-way ANOVA with Holm-Sidak post hoc correction to test for the effect of lipopolysaccharide (LPS), *P2rx7* knockout (Genotype), and the interaction. For all analyses, P<0.05 was considered significant.

**Supplemental Figure 5: *Ex vivo* renal artery contractility in female *P2rx7^-/-^* rats**

A) Similar external K^+^-evoked constriction force in female WT and *P2rx7^-/-^* rat renal artery (n=8 rats/genotype). B) Similar vasoconstriction of female WT and *P2rx7^-/-^* rat renal arteries to increasing phenylephrine (PE) concentrations (n=8 rats/genotype). C) Similar vasodilation of female *P2rx7^-/-^* rat renal arteries to increasing acetylcholine (ACh) concentrations (n=8 rats/genotype). D) Slightly enhanced vasodilation of female *P2rx7^-/-^* rat renal arteries to increasing sodium nitroprusside (SNP) concentrations (n=8 rats/genotype). Data are means ± SD and statistical analysis performed using t-test (A) or 2-way ANOVA (B to D). For all analyses, P<0.05 was considered significant.

**Supplemental Figure 6: Effect of P2X7 antagonist AZ11657312 on renal artery vasoconstriction in male WT and *P2rx7^-/-^* rats**

A) Effect of P2X7 antagonist AZ11657312 (10μM) on phenylephrine-evoked renal artery vasoconstriction in male WT rats (n=4 rats, 2 arteries per rat). B) Effect of P2X7 antagonist AZ11657312 (10μM) on phenylephrine-evoked renal artery vasoconstriction in male *P2rx7^-/-^* rats (n=4 rats, 2 arteries per rat). Data are means ± SD and statistical analysis performed using 2-way ANOVA. For all analyses, P<0.05 was considered significant.

**Supplemental Figure 7: *In vivo* renal hemodynamics and the pressure natriuresis relationship in female *P2rx7^-/-^* rats**

A) Change in mean arterial pressure (MAP) following ligation of coeliac, superior mesenteric, and distal aorta ligation. B) Change in renal artery blood flow (RBF) as measured using a Transonic Doppler flow probe placed around the right main renal artery. C) Change in renal vascular resistance (RVR). D) Change in glomerular filtration rate (GFR). E) Change in urinary sodium excretion rate (U_Na_V). F) Change in urine flow rate (UV). For all, n=9-10 rats/group. Data are means ± SD and statistical analysis performed using t-test. For all analyses, P<0.05 was considered significant.

**Supplemental Figure 8: Blood pressure parameters in anesthetized *P2rx7^-/-^* rats**

Change in systolic (A) and diastolic (B) blood pressure, and heart rate (C) following ligation of coeliac, superior mesenteric, and distal aorta ligation in anesthetized female WT and *P2rx7^-/-^* rats. Change in systolic (D) and diastolic (E) blood pressure, and heart rate (F) following arterial ligation in anesthetized male WT and *P2rx7^-/-^* rats. For all, n=9-10 rats/group. Data are means ± SD and statistical analysis performed using t-test. For all analyses, P<0.05 was considered significant.

**Supplemental Figure 9: Renal hemodynamic parameters in anesthetized**

***P2rx7^-/-^* rats**

Change in renal cortical (A) and medullary (B) blood perfusion following ligation of coeliac, superior mesenteric, and distal aorta ligation in anesthetized female WT and *P2rx7^-/-^* rats. Change in renal cortical (C) and medullary (D) blood perfusion following arterial ligation in anesthetized male WT and *P2rx7^-/-^* rats. For all, n=9-10 rats/group. Data are means ± SD and statistical analysis performed using t-test. For all analyses, P<0.05 was considered significant.

**Supplemental Figure 10: Urinary potassium and chloride excretion in anesthetized *P2rx7^-/-^* rats**

Change in urinary potassium (A) and chloride (B) excretion following ligation of coeliac, superior mesenteric, and distal aorta ligation in anesthetized female WT and *P2rx7^-/-^* rats. Change in urinary potassium (C) and chloride (D) excretion following arterial ligation in anesthetized male WT and *P2rx7^-/-^* rats. For all, n=9-10 rats/group. Data are means ± SD and statistical analysis performed using t-test. For all analyses, P<0.05 was considered significant.

**Supplemental Figure 11: Vascular remodelling in heart of male *P2rx7^-/-^* rats following chronic ANGII infusion**

A) Representative heart cross-sections of male WT and *P2rx7^-/-^* rats following chronic ANGII infusion stained with picrosirius red for collagen fibres. Scale bar = 1000μm. B) Representative images of blood vessels in myocardium of WT and *P2rx7^-/-^* rats following ANGII infusion, or naive controls. Scale bar = 100μm. Quantification of perivascular collagen area in heart sections. For all, n=6 rats/group. Data are means ± SD and statistical analysis performed using 2-way ANOVA with Holm-Sidak post hoc correction to test for the effect of angiotensin II (ANGII), *P2rx7* knockout (Genotype), and the interaction. For all analyses, P<0.05 was considered significant.

**Supplemental Figure 12: *Ex vivo* renal artery contractility in male *P2rx7^-/-^* rats following chronic ANGII infusion**

A) Similar external K^+^-evoked constriction force of renal arteries from naïve rats and rats chronically infused with ANGII in both WT and *P2rx7^-/-^* (n=9-11 rats/group). B) Similar vasodilation to increasing acetylcholine (ACh) concentrations of renal arteries from naïve rats and rats chronically infused with ANGII in both WT and *P2rx7^-/-^* (n=9-11 rats/group). (n=8 rats/genotype). C) Similar vasodilation to increasing sodium nitroprusside (SNP) concentrations of renal arteries from naïve rats and rats chronically infused with ANGII in both WT and *P2rx7^-/-^* (n=9-11 rats/group). Data are means ± SD and statistical analysis performed using t-test (A) or 2-way ANOVA (B and C). For all analyses, P<0.05 was considered significant.
